# Simultaneous Determination and Pharmacokinetics Study of Six Triterpenes in Rat Plasma by UHPLC-MS/MS after Oral Administration of *Sanguisorba officinalis* L. Extract

**DOI:** 10.3390/molecules23112980

**Published:** 2018-11-15

**Authors:** Chengcui Wu, Meicun Yao, Wa Li, Binbin Cui, Hongrui Dong, Yixuan Ren, Chunjuan Yang, Chunli Gan

**Affiliations:** 1 Department of Pharmaceutical Analysis and Analytical Chemistry, College of Pharmacy, Harbin Medical University, No. 157 Baojian Road, Nangang District, Harbin 150081, China; 18845645250@163.com (C.W.); binbincui0419@163.com (B.C.); donghongrui422@163.com (H.D.); renyixuan1218@163.com (Y.R.); 2 School of Pharmaceutical Sciences, Sun Yat-sen University, Guangzhou 510006, China; yaomeicun@gmail.com (M.Y.), wa1134055927@163.com (W.L.); 3 Department of Medicinal Chemistry and Natural Medicine Chemistry, College of Pharmacy, Harbin Medical University, No. 157 Baojian Road, Nangang District, Harbin 150081, China

**Keywords:** ultra-high-performance liquid chromatography with tandem mass spectrometry, triterpenes, *Sanguisorba officinalis* L., pharmacokinetics

## Abstract

A selective and sensitive ultra-high-performance liquid chromatography-tandem mass spectrometry (UHPLC-MS/MS) method was developed and validated for the determination of ziyuglycoside I (**I**), 3β,19α-dihydroxyurs-12-en-28-oic-acid 28-β-d-glucopyranosyl ester (**II**), 3β-[(α-l-arabinopyranosyl) oxy]-urs-12,18(19)-dien-28-oic acid β-d-glucopyranosyl ester (**III**), rosamultin (**IV**), 1β-hydroxyeuscaphic acid (**V**) and alpinoside (**VI**) in rats after oral administration of *Sanguisorba officinalis* L. (*S. officinalis*) extract. The 3β,19α-dihydroxyurs-12-en-28-oic-acid 28-β-d-glucopyranosyl ester, 3β-[(α-l-arabinopyranosyl) oxy]-urs-12,18(19)-dien-28-oic acid β-d-glucopyranosyl ester, rosamultin, 1β-hydroxyeuscaphic acid and alpinoside in rat plasma were the first report in the pharmacokinetics study in the present study. The analytes were quantified using the multiple reaction monitoring (MRM) mode with the electrospray ion source in positive electrospray ionization. Plasma was extracted with ethyl acetate via liquid–liquid extraction. Bifendate was used as internal standard (IS). The current method was validated for linearity, intra-day and inter-day precisions, accuracy, extraction recovery, matrix effect and stability. The lower limits of quantification of ziyuglycoside I, 3β,19α-dihydroxyurs-12-en-28-oic-acid 28-β-d-glucopyranosyl ester, 3β-[(α-l-arabinopyranosyl) oxy]-urs-12,18(19)-dien-28-oic acid β-d-glucopyranosyl ester, rosamultin, 1β-hydroxyeuscaphic acid and alpinoside were 6.1, 4.9, 1.3, 3.8, 1.5 and 5.7 ng/mL, respectively. Intra-day and inter-day precision and the accuracy of the assay were in range from −9.48 to 12.74%. The extraction recoveries of analytes and bifendate (IS) from rat plasma ranged from 77.17% to 92.48%. Six compounds could be rapidly absorbed into blood (*T_max_*, 0.58–1.58 h), and then eliminated relatively slowly (*t*_1/2_, 6.86–11.63 h). The pharmacokinetic results might contribute to further guide the clinical application of *S. officinalis*.

## 1. Introduction

*Sanguisorba officinalis* L. *(S. officinalis)*, which is a traditional Chinese medicine (TCM), and a member of Rosaceae family, has the effects of detoxification, analgesic [1] and hemostatic [2]. According to the Chinese pharmacopoeia, it plays a major role in the treatment of hematochezia, bleeding hemorrhoids, bloody flux, metrorrhagia and metrostaxis, bleeding wounds, burns and scalds, and swollen carbuncles [3]. Besides, in vivo and in vitro studies have illustrated that plants from the *S. officinalis* present a wide range of pharmacological properties, including hemostatic, antioxidant [4], anti-inflammatory [5], antiviral [6], antibacterial [7,8], anti-tumor [9], neuroprotective and hypoglycemic activities [10]. Simultaneously, in Chinese medical practice, many drugs (e.g., tablets and powders) that contain *S. officinalis* roots have been applied to treat leukopenia, hemorrhaging and burns [11]. It has been reported that *S. officinalis* has obvious anti-tumor effect, which inhibits the growth of human leukemia cell K562, hepatoma cell HepG2, gastric cancer cell BGC823, leukemia cell L1210, cervical cancer cell Hela, and lung cancer cell H460, and induces apoptosis of human liver cancer SMMC-7721 cells [12,13,14,15]. The main chemical constituents isolated from *S. officinalis* include triterpenes and their glycosides [16,17], tannins, flavonoids [2,17], etc. Triterpenes are the main hemostatic components of *S. officinalis,* the pharmacological studies mainly focus on antioxidant, anti-inflammatory and anti-tumor activities [2,18,19] in nearly a decade.

**Figure 1:**
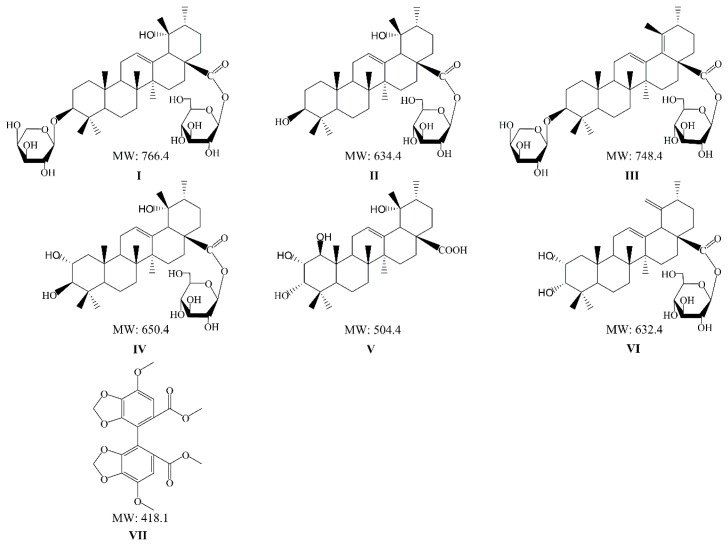
Chemical structures of: ZGI (**I**); DGE (**II**); AGE (**III**); RMU (**IV**); HDA (**V**); APS (**VI**); and IS (**VII**).

Ziyuglycoside I (ZGI) (**I**), 3β,19α-dihydroxyurs-12-en-28-oic-acid 28-β-d-glucopyranosyl ester (DGE) (**II**), 3β-[(α-l-arabinopyranosyl) oxy]-urs-12,18(19)-dien-28-oic acid β-d-glucopyranosyl ester (AGE) (**III**), rosamultin (RMU) (**IV**), 1β-hydroxyeuscaphic acid (HDA) (**V**) and alpinoside (APS) (**VI**) (Figure 1) are the active components of triterpenes isolated from *S. officinalis*, and many studies have concentrated upon their pharmacological properties [20]. Six triterpenes have not only common pharmacological activities, but also their own pharmacological characteristics. On the one hand, ZGI, one of the main triterpenes in *S. officinalis*, was considered to play a role in eliminating free radicals and inhibiting elastase activity [21,22]. On the other hand, ZGI could inhibit skin wrinkles by boosting the production of collagen, not by its anti-oxidant activity [23]. It has been reported that DGE significantly inhibited NO production [24]. The effects of DGE on reduction of both d-galactosamine (d-GalN) and TNF-α-induced cytotoxicity, the viability of L929 cells and a TNF-α-sensitive cell line were examined under the presence of the constituents [25]. Thus, DGE significantly improved cell viability. Some studies have shown that RMU possesses antioxidant and anti-apoptosis effects, which could treat H_2_O_2_-induced oxidative stress injury [26]. HDA, which is one of triterpenes ingredients, could effectively attenuate the leakage of intracellular enzymes, and decrease the oxidation of proteins and the incidence of apoptosis. Thus, its remarkable hepatoprotective effect was revealed [27].

Triterpenes have made great progress in pharmacological research, but there are few studies on pharmacokinetic aspects. Several methods have been applied to the determination of triterpenes in the past few years. For instance, Ye et al. offered an original and universally appropriate method to determine ziyuglycoside I and ziyuglycoside II in rat plasma based on LC-MS [22]. Besides, in 2018, Li et al. developed a simple and sensitive HPLC-MS/MS method for simultaneous determination and pharmacokinetics of ziyuglycoside I and its metabolite ziyuglycoside II in rats [28]. However, there are few reports on the simultaneous determination and pharmacokinetics of ZGI, DGE AGE, RMU, HDA and APS from *S.* *officinalis.* Thus, the pharmacokinetics of DGE, AGE, RMU, HDA and APS are reported for the first time in this paper.

Hence, the purpose of this study was to set up a sensitive and efficient UHPLC-MS/MS method for simultaneous determination and pharmacokinetics of six analytes in rats after single oral administration of *S. officinalis* extract. Meanwhile, the DGE, AGE, RMU, HDA and APS are the first report in pharmacokinetic study of *S. officinalis*. This study could be conducive to furnish basis for clinical application of *S. officinalis*. 

## 2. Results 

### 2.1. UHPLC-MS/MS Optimization

**Figure 2:**
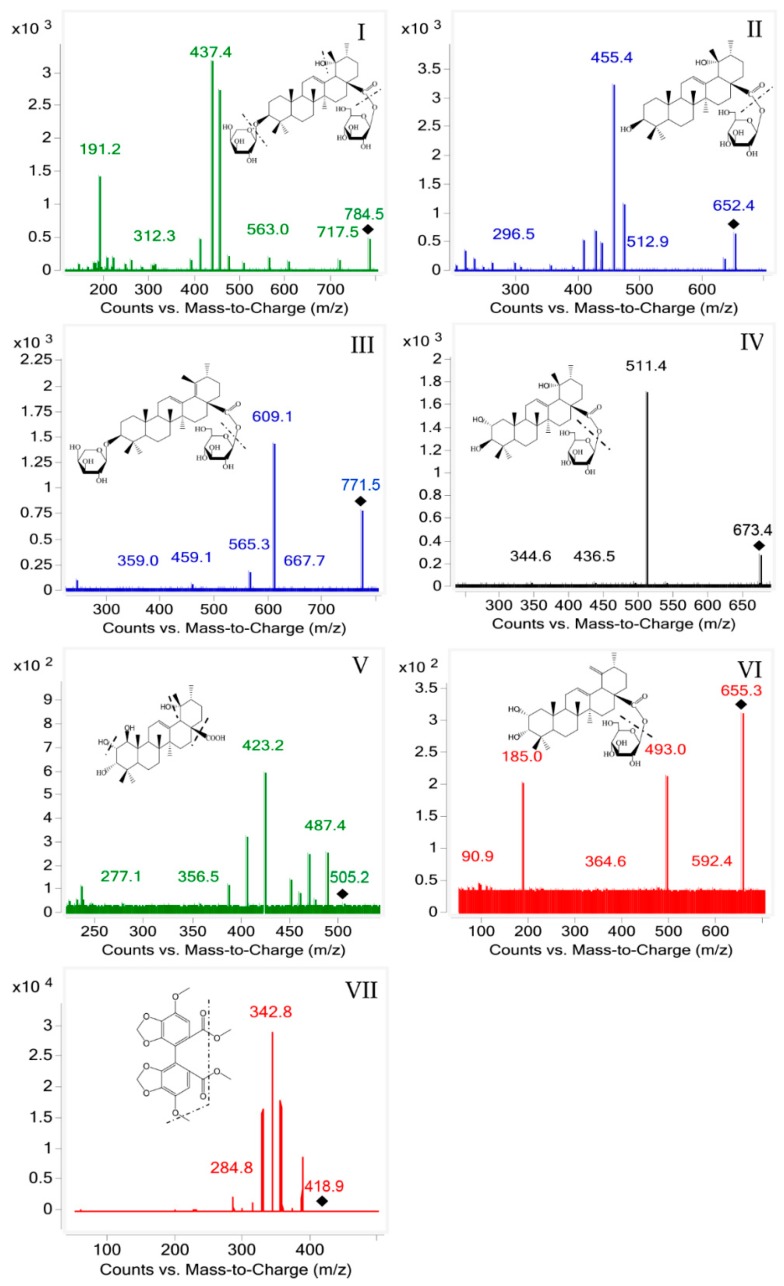
Product ion mass spectra of: ZGI (**I**); DGE (**II**); AGE (**III**); RMU (**IV**); HDA (**V**); APS (**VI**); and IS (**VII**).

The analysis was performed on an Agilent series 1290 UHPLC instrument coupled with an Agilent Technologies 6430 mass spectrometer with an electrospray ionization (ESI) interface. The eluent was monitored using a triple quadrupole tandem mass spectrometer equipped with ESI source and operated in positive ion mode with MRM. The precursor ion was [M + NH_4_]^+^ at *m*/*z* 784.5 Da for **I**, and the product ion peak at *m*/*z* 437.4 Da was attributable to one molecule of C_5_H_9_O_4_ (133 Da), one molecule of C_6_H_11_O_6_ (179 Da) and two molecules of H_2_O loss (36 Da). The precursor ion was [M + NH_4_]^+^ at *m*/*z* 652.5 Da for **II**, and the product ion peak at *m*/*z* 455.4 Da was attributable to one molecule of C_6_H_11_O_6_ (179 Da) and one molecule of H_2_O loss (18 Da). The precursor ion was [M + Na]^+^ at *m*/*z* 771.5 Da for **III**, and the product ion peak at *m*/*z* 609.1 Da was attributable to one molecule of C_6_H_11_O_5_ (163 Da). The precursor ion was [M + Na]^+^ at *m*/*z* 673.4 Da for **IV**, and the product ion peak at *m*/*z* 511.4 Da was attributable to one molecule of C_6_H_11_O_5_ (163 Da). The precursor ion was [M + Na]^+^ at *m*/*z* 505.2 Da for **V**, and the product ion peak at *m*/*z* 423.2 Da was attributable to one molecule of COOH (45 Da) and two molecules of H_2_O loss (36 Da). The precursor ion was [M + Na]^+^ at *m*/*z* 655.4 Da for **VI**, and the product ion peak at *m*/*z* 493.0 Da was attributable to one molecule of C_6_H_11_O_5_ (163 Da). The precursor ion was [M + H]^+^ at *m*/*z* 418.9 Da for **VII**, and the product ion peak at *m*/*z* 342.8 Da was attributable to one molecule of CH_3_ (15 Da) and two molecules of OCH_3_ loss (62 Da). The mass parameters for six analytes and IS are summarized in Table 1. Chemical structure and product ion scan spectra of six compounds and IS are presented in Figure 2.

### 2.2. Method Validation

#### 2.2.1. Extraction Recovery and Matrix Effect

The extraction recoveries and matrix effects of six compounds from rat plasma are presented in Table 2. In this experiment, the use of ethyl acetate as extraction solvent has an excellent extraction efficiency. The extraction recoveries of six analytes in rat plasma were 77.17–91.80% at three QC levels, respectively. The extraction recovery of IS was 92.48%. The matrix effects ranged from 100.17% to 102.10% for six compounds at low, medium, and high QC levels in rat plasma. No significant matrix effects affecting the six analytes were detected in rat plasma.

#### 2.2.2. Linearity and LLOQ

As shown in Table 3, the calibration curves for six compounds have good linearity over the concentration ranges of 6.1–2420 ng/mL (**I**), 4.9–1950 ng/mL (**II**), 1.3–533.3 ng/mL (**III**), 3.8–1510 ng/mL (**IV**), 1.5–604.0 ng/mL (**V**) and 5.7–2250 ng/mL (**VI**) with all correlation coefficients (*r*) > 0.9912. The typical equations of calibration curves are listed in Table 3, where *X* means the plasma concentration of analytes and *Y* represents the peak area ratio of analytes to IS. The results showed that compounds are within the good linearity ranges. The LLOQs of **I**–**VI** were 6.1, 4.9, 1.3, 3.8, 1.5 and 5.7 ng/mL, respectively.

#### 2.2.3. Selectivity

**Figure 3:**
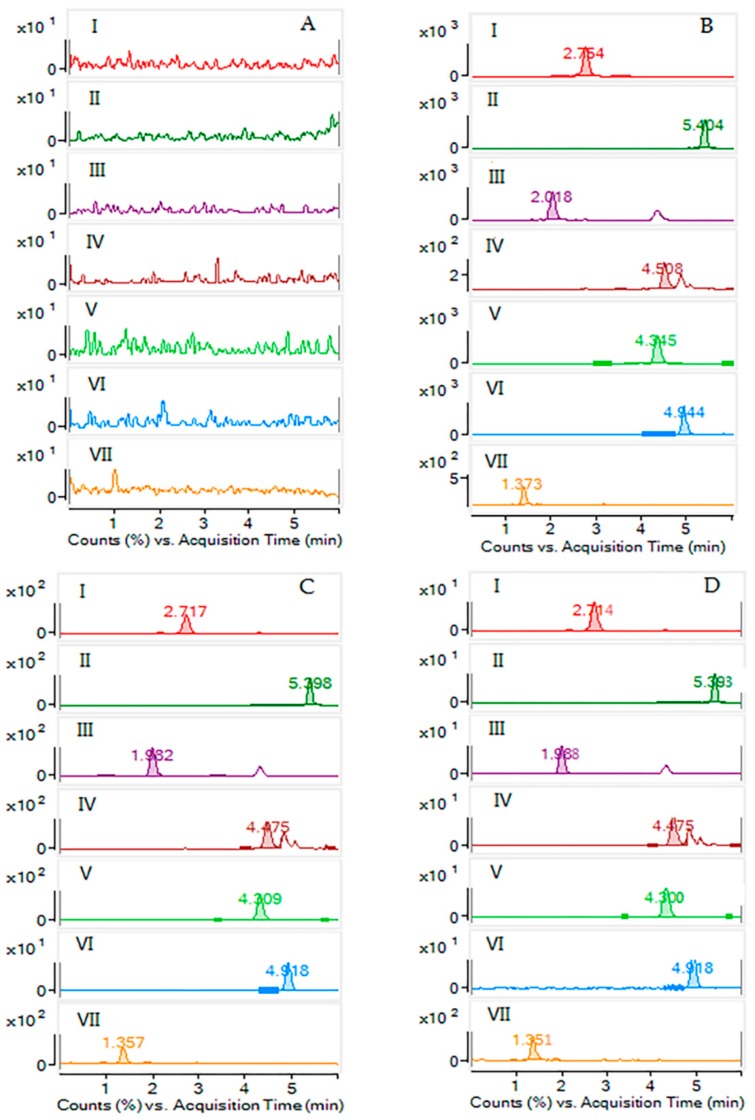
The chromatograms of: ZGI (**I**); AGE (**II**); RMU (**III**); APS (**IV**); DGE (**V**); HDA (**VI**); and IS (**VII**) in rat plasma samples. (**A**) Blank plasma; (**B**) plasma samples after oral administration of *S. officinalis* extract 0.5 h; (**C**) LLOQ sample (six analytes and IS in blank plasma); and (**D**) the blank plasma with QCM analytes and IS (121.0 ng/mL for ZGI, 97.5 ng/mL for DGE, 26.7 ng/mL for AGE, 75.5 ng/mL for RMU, 30.2 ng/mL for HDA, and 112.5 ng/mL for APS).

The selectivity of six analytes was evaluated via comparing the chromatograms of blank plasma, plasma sample spiked with LLOQ analytes and IS, blank plasma with QCM analytes and IS, and the plasma sample from rats following single oral administration of *S. officinalis* extract (Figure 3). All results demonstrate that no endogenous substances interfered at the retention time of IS and analytes. 

#### 2.2.4. Precision and Accuracy

Precisions and accuracies of six analytes in rat plasma at LLOQ, LQC, MQC, and HQC levels are listed in Table 4. The intra-day and inter-day precisions (RSD) of six compounds were all less than 12.74%, and the accuracies (RE) were from −9.48% to 10.15% for six analytes. Precision and accuracy conformed to relevant rules for the guidance of biological samples analysis [29]. 

#### 2.2.5. Stability

The stability of six analytes in rat plasma was estimated under different storage conditions. The results (Table 5) demonstrate that six analytes in rat plasma were steady after three freeze–thaw cycles, at room temperature for 4 h. Post-preparative stability of analytes also implied that there was no obvious degradation when samples were kept at 4 °C for 12 h. In addition, all compounds remained stable for two weeks at −20 °C.

### 2.3. Pharmacokinetic Studies

**Figure 4:**
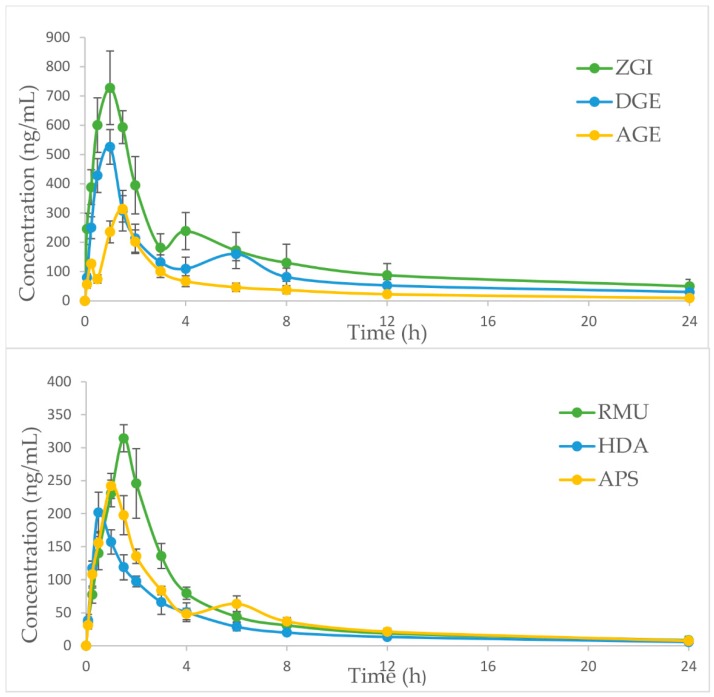
Mean plasma concentration-time curve of six analytes in rats after oral administration of *S. officinalis* extract (*n* = 12, mean ± SD).

The UHPLC-MS/MS method was successfully used in the pharmacokinetic studies of six analytes after single dose administration of *S. officinalis* extract in rats. Based on the body surface area calculations of people and the animals and equivalent dose conversion calculations, the dosage of the rats was 0.015 g/kg [30]. The mean plasma concentration–time curves of compounds are listed in Figure 4. The half-time (*t*_1/2_), maximum plasma concentration (*C_max_*), time to reach *C_max_* (*T_max_*), and area under concentration–time curve (AUC_0__→t_ and AUC_0__→__∞_) were calculated via non-compartmental analysis (Table 6). The pharmacokinetic parameters of six components were reckoned by means of non-compartmental analysis using DAS 2.0 (Shanghai, China).

## 3. Discussion

Even though scholars have employed the LC-MS/MS method, the selective ion monitoring mode (SIM) is mainly used to take the place of MRM mode, there may be more interference in the SIM mode [31]. Therefore, a sensitive and efficient UHPLC-MS/MS method was established for simultaneous determination of the six analytes in MRM mode. 

It is vital for the optimization of mass spectrometry parameters to acquire steady and sensitive responses for analytes. The analysis was performed on an Agilent series 1290 UHPLC instrument (Agilent Technologies, Santa Clara, CA, USA) coupled with an Agilent Technologies 6430 mass spectrometer (Agilent Technologies, Santa Clara, CA, USA) with an electrospray ionization (ESI) interface. The eluent was monitored using a triple quadrupole tandem mass spectrometer (Agilent Technologies, Santa Clara, CA, USA) equipped with ESI source and operated in positive ion mode with MRM. To achieve good resolution, several different chromatographic columns including a XTerra^®^ MS C_18_ (2.1 × 50 mm, 2.5 μm, Waters Technologies, Milford, MA, USA) column, an ACQUITY UPLC^®^ HSS T3 (2.1 × 100 mm, 1.8 μm, Waters Technologies, Milford, MA, USA) column, and an Agilent Eclipse Plus C_18_ RRHD (2.1 × 50 mm, 1.8 μm, Agilent Technologies, Santa Clara, CA, USA) column were attempted. Finally, the Agilent Eclipse Plus C_18_ RRHD (2.1 × 50 mm, 1.8 μm, Agilent Technologies, Santa Clara, CA, USA) column was adopted to achieve great resolution. In summary, for the sensitive detection of six analytes, the positive mode was adopted with MRM. The fragment and collision energy were optimized to increase the sensitivity of the six analytes. The conditions of MS analysis are as follows: drying gas (N_2_) flow-rate, 11 L/min; drying gas temperature, 300 °C; high purity nitrogen (N_2_) was atomized as the nebulizing gas; and capillary voltage, 4000 V. 

Chromatographic conditions were updated to meliorate peak shape, enhance signal response of six components and reduce the running time. We attempted different mobile phase systems including acetonitrile–water, and methanol–water in terms of different proportions. When methanol–water was used as mobile phase, the response of six analytes was apparently higher than that of acetonitrile–water. Various additives have a notable impact on improving the response of analytes. Different additives such as formic acid (0.1%), ammonium acetate (2 and 5 mM) and acetic acid (0.1%) were investigated. Finally, 0.1% formic acid water was selected as mobile phase to increase the peak intensity of six analytes. It is worth noticing that the high resolution of the UHPLC system increases the speed and peak capacity of the six analytes. At the same time, there was no crosstalk by adjusting all aspects. Finally, the optimized separating conditions were obtained with methanol–0.1% formic acid water as mobile phase at 30 °C and a flow rate of 0.3 mL/min. The mobile phase was as follows: solvent A was 0.1% formic acid water, and solvent B was methanol, which was delivered at a flow rate of 0.3 mL/min. The gradient elution program was as follows: 0–4.0 min, 60% to 65% B; 4.0–4.5 min, 65% to 90% B; 4.5–5.6 min, 90% B; 5.6–6.0 min, 90% to 60% B. The all run time was 6.0 min, and sample injection volume was 5 μL. 

To achieve high extraction recovery and weak matrix effect, it is crucial for simultaneously and accurately analyzing the target compound to choose a reasonable sample preparation method [32]. For sample preparation, we have tried some extraction methods such as SPE (Solide Phase Extraction), protein precipitation and LLE. We tried the protein precipitation method due to its simplicity. Nevertheless, the drawback is that the recovery of compounds is not only insufficient but also non-renewable. Moreover, SPE is time-consuming and columns are relatively expensive. Thus, LLE was selected as sample preparation method owing to its constant extraction recoveries, and negligible matrix effects. It is important for the extraction recoveries to choose a suitable organic solvent as the extract agent. Thus, several solvents have been attempted including ether, dichloromethane and ethyl acetate. Because six analytes have similar polarities, ethyl acetate is the best choice in terms of extraction efficiency and reproducibility.

It is important for the pharmacokinetic study to select reasonable internal standard. The IS should be provided with similar polarity and solubility, it should not react with analytes, and it should not interfere with compounds. In this experiment, we tested bifendate and theophylline. Eventually, bifendate was chosen as IS, which has the suitable retention time and good precision in this experiment. Meanwhile, bifendate had no interference with the analytes and may be utilized to determine the concentration of triterpenes.

As shown in Table 6, the pharmacokinetic process of the six analytes in *S. officinalis* extract was different. The *T_max_* values of ZGI, DGE, AGE, RMU, HDA and APS were 0.92 ± 0.20, 0.83 ± 0.26 and 1.33 ± 0.26, 1.58 ± 0.20, 0.58 ± 0.20 and 1.17 ± 0.26 h, respectively, after single dose administration of *S. officinalis* extract, which indicated the absorbance velocity of six compounds was relatively rapid. The *C_max_* values of ZGI, DGE, AGE, RMU, HDA and APS in *S. officinalis* extract were 744.66 ± 85.74, 526.58 ± 64.02, 317.87 ± 47.60, 314.53 ± 22.46, 211.51 ± 11.81 and 243.21 ± 19.90 ng/mL, respectively. It may be attributed to the difference in the content of six analytes in *S. officinalis* extract. Furthermore, as report goes, many herbal medicine or natural compounds separated from Chinese medicinal materials have been appraised as substrates, inhibitors and inducers of various CYP3A4, and herb–CYP interactions, the above-mentioned illustrated that it was possible to have impact on the pharmacokinetics of some compounds [33]. Moreover, the *t*_1/2_ of ZGI, DGE, AGE, RMU, HDA and APS were 11.63 ± 3.07, 8.99 ± 3.04, 6.86 ± 2.91, 7.32 ± 2.74, 7.35 ± 3.95 and 7.72 ± 1.22 h, respectively. It was revealed that the other five compounds were eliminated and metabolized quickly compared with ZGI. The slow elimination of ZGI may be attributed to its high content in *S. officinalis* extract. Besides, according to our research results, the *t*_1/2_ of ZGI was 11.63 h instead of 19.76 or 6.12 h, which is different from the *t*_1/2_ of ZGI in the reported literature [22,28]. The difference of *t*_1/2_ values of the ZGI may be caused by the different dosage, the way of administration and the complexity of Chinese medicine composition. Moreover, the AUC_0→t_ values of six compounds in *S. officinalis* extract were 2879.96 ± 303.36, 2296.46 ± 416.63, 1139.15 ± 150.38, 1208.39 ± 119.71, 733.31 ± 94.08 and 1026.03 ± 73.43 ng·h/mL, respectively. The AUC_0→∞_ values of six compounds were 3319.05 ± 429.07, 2661.61 ± 600.92, 1231.82 ± 192.74, 1302.86 ± 192.89, 794.41 ± 151.77 and 1112.72 ± 98.12 ng·h/mL, respectively. As shown in Figure 4, the mean plasma concentration–time distribution curves of ZGI, DGE, AGE and APS exhibited the double-peak phenomenon during elimination phase. The first peak of ZGI, DGE and APS, which occurred at 0.5–1.5 h, the second one appeared at 4-8 h after oral administration. Compared with three compounds, the first peak of AGE appeared in 0.25 h, and the second emerged before 1.5 h. The second peak was far greater than the first peak. The double-peak phenomenon of compounds may be due to the distribution of reabsorption and entero-hepatic circulation [34]. These results could be conducive to further explore the mechanism of triterpenes and provide effective pharmacokinetic information for *S. officinalis*.

## 4. Materials and Methods

### 4.1. Chemicals and Reagents

The ZGI, DGE, AGE, RMU, HDA and APS, the purities of which were more than 98%, were refined in our laboratory (identified by NMR and MS). Bifendate (lot: 73536-69-3; purity > 98%, IS) was purchased from Chengdu Must Bio-Technology (Chengdu, Sichuan province, China). Methanol and acetonitrile (HPLC-grade) were obtained from J & K Medical (Beijing, China). Ammonium acetate was purchased from Kermel (Tianjin, China). Ultra-pure water was gained by using Milli-Q water purification system (Millipore, Molsheim, France). All other reagents including ethyl acetate, ether and dichloromethane were of analytical grade. The plasma samples were obtained from the blood of rat. 

The *S. officinalis* was collected from the Anguo Traditional Chinese Medicine Market of Hebei and authenticated by Professor Zhenyue Wang of Heilongjiang University of Chinese Medicine in September 2016. A voucher specimen was deposited in Pharmaceutical Research Department of Harbin Medical University, China.

### 4.2. Preparation of S. officinalis Extract

After crushing the dried root of *S. officinalis* (200 g), it was extracted by hot reflux with 2 L 70% ethanol (1:10, *w*/*v*) solution 2 times at 80 °C, 60 min each, and then filtrated. The combined filtrate was evaporated to steam, and the residue was dissolved in water to get a concentration equivalent to 0.05 g/mL of the *S. officinalis* extract [35]. The contents of *S. officinalis* extract for **I**–**VI** were 50.26, 33.20, 18.01, 11.34, 22.34 and 6.91 mg/g, respectively. The results of simultaneous determination of six triterpenes from *S. officinalis* extract by HPLC-ELSD are presented in the Supplementary Materials.

### 4.3. Preparation of Calibration Standards and QC Samples

Standard stock solutions of **I**–**VI** were gained through dissolving each compound in methanol to yield a nominal concentration (0.24 mg/mL, 0.11 mg/mL, 0.13 mg/mL, 0.14 mg/mL, 0.13 mg/mL, and 0.13 mg/mL, respectively). Standard working solutions were prepared by appropriate dilutions of the stock solutions with methanol (6.1–2420 ng/mL for **I**, 4.9–1950 ng/mL for **II**, 1.3–533.3 ng/mL for **III**, 3.8–1510 ng/mL for **IV**, 1.5–604.0 ng/mL for **V**, and 5.7–2250 ng/mL for **VI**). The IS stock standard solution was diluted to a 1040 ng/mL working solution. Calibration standards were prepared by spiking each working stock solution at seven concentrations of: 6.1, 12.1, 24.2, 121.0, 242.0, 484.0 and 2420 ng/mL for **I**; 4.9, 9.8, 19.5, 97.5, 195.0, 390.0 and 1950 ng/mL for **II**; 1.3, 2.7, 5.3, 26.7, 53.4, 106.7 and 533.3 ng/mL for **III**; 3.8, 7.6, 15.1, 75.5, 151.0, 302.0 and 1510 ng/mL for **IV**; 1.5, 3.0, 6.0, 30.2, 60.4, 120.8 and 604.0 ng/mL for **V**; and 5.7, 11.3, 22.5, 112.5, 225.0, 450.0 and 2250 ng/mL for **VI**. Quality control (QC) samples were prepared at: 12.1, 121.0 and 1936 ng/mL for **I**; 9.8, 97.5 and 1560 ng/mL for **II**; 2.7, 26.7 and 426.67 ng/mL for **III**; 7.6, 75.5 and 1208 ng/mL for **IV**; 3.0, 30.2 and 483.2 ng/mL for **V**; and 11.3, 112.5, and 1800 ng/mL for **VI**. LLOQ of **I**–**VI** was 6.1, 4.9, 1.3, 3.8, 1.5 and 5.7 ng/mL, respectively. All solutions were immediately stored at 4 °C.

### 4.4. Animals Experiments 

The experimental protocol was permitted by the Animal Ethics Committee of Harbin Medical University and conformed to the principles for the Care and Use of Laboratory Animals. Twelve male Sprague-Dawley rats (Weight 200 ± 20 g) were provided by the Laboratory Animal Centre of Harbin Medical University (Harbin, China). Each rat was fasted for 12 h before giving the drug and had free water supply even during the experiment. The *S. officinalis* extract was dissolved in water. A single dose of the *S. officinalis* extract (0.015 g/kg) was administrated to the rats. Blood (0.3 mL) was gained from the retinal venous plexus at 0, 0.083, 0.25, 0.5 1.0, 1.5, 2.0, 3.0, 4.0, 6.0, 8.0, 12.0 and 24.0 h after dosing. The plasma was immediately separated by centrifugation at 12000 rpm for 5 min at −4 °C.

### 4.5. Plasma Samples Preparation

Ten microliters of IS (1040.0 ng/mL) solution and 100 μL of methanol were added to 100 μL aliquot of plasma sample and vortexed for 30 s. The mixture was extracted with 3 mL ethyl acetate by being vortex-mixed for 1 min. The supernatant was separated and evaporated to dryness by N_2_ blowing at 40 °C after centrifuging at 3800 rpm for 5 min. The residue was reconstituted with 100 μL of methanol, and then vortex-mixed for 2 min and filtered by a 0.22 μm nylon 66 organic membrane. This was followed by injection of 5 μL aliquot of the solution into the UHPLC-MS/MS system (Agilent Technologies, Santa Clara, CA, USA) [36]. 

### 4.6. Method Validations

The selectivity, linearity, precision, accuracy, extraction recovery, matrix effect and stability were evaluated based on the FDA guidelines [29]. 

#### 4.6.1. Selectivity 

The method of selectivity was used in the quantitative analysis of possible interfering substances in samples; the results show that this method was accurate and specific. All results demonstrate that no endogenous substances interfered with quantitative analysis.

#### 4.6.2. Recovery and Matrix Effect

The extraction recovery of analytes was determined via comparing the peak areas of the six analytes from the QC samples with those obtained from blank plasma samples with the six analytes spiked into the post-extraction supernatant at three QC levels in six replicates. The matrix effect was evaluated through comparing the peak areas of analytes spiked after plasma extraction with those of standard samples. The extraction recovery and matrix effects of IS were also measured at one concentration.

#### 4.6.3. Linearity and LLOQ

The calibration curves were constructed by plotting the peak area ratio versus the concentration of the six analytes and IS with a weighted (1/*x*^2^) least square linear regression using standard plasma samples. The lower limit of quantification (LLOQ) was defined as the lowest analytical concentration of the calibration curve with an acceptable precision (RSD) below 20% and accuracy (RE) within ±20%. The lower limit of detection (LLOD) was determined as the concentration of the analytes with a signal-to-noise ratio at 3 in the blank plasma.

#### 4.6.4. Precision and Accuracy

The intra- and inter-day precision and accuracy were measured by testing the LLOQ sample and QC samples at three QC levels of six compounds in six replicates on three days in a row. The precision was determined and expressed as RSD and the accuracy as relative error (RE). The intra-day and inter-day precision and accuracy were within 15%, which is an acceptable requirement. The RSD of LLOQ samples should be within 20%.

#### 4.6.5. Stability 

The stability of six compounds in rat plasma including freeze and thaw stability (three freeze–thaw cycles at −20 °C), long-term stability (storage for 2 weeks at −80 °C), room temperature stability (storage for 4 h at ambient temperature), and post-preparation stability (storage for 12 h after sample preparation at 4 °C) was tested at three QC levels with five replicates at each level. All stability testing QC samples were determined by using the calibration curve of freshly prepared standard samples.

### 4.7. Plasma Pharmacokinetic Study

The maximum concentration (*C_max_*) and the time to attain it (*T_max_*) were observed directly from the measured data. The elimination rate constant (*K_e_*) was calculated by linear regression of the terminal points in a semi-log plot of the plasma concentration against time. The elimination half-life (*t*_1/2_) was calculated using the formula *t*_1/2_ = 0.693/*K_e_*. The area under plasma concentration–time curve (AUC_0→t_) to the last measurable plasma concentration (*C_t_*) was estimated by using the linear trapezoidal rule. The area under the plasma concentration–time curve to time infinity (AUC_0→__∞_) was calculated as: AUC_0→__∞_ = AUC_0→t_ + C_t_/*K_e_*. The pharmacokinetic parameters of six analytes were reckoned by non-compartmental analysis using DAS 2.0 (Mathematical Pharmacology Professional Committee of China, Shanghai, China).

## 5. Conclusions

This study developed a simple, rapid and sensitive LC-MS/MS method for simultaneous quantification of six components from *S. officinalis* in rat plasma. Based on literature review, this is the first report of pharmacokinetic study of six triterpenes together in vivo following the oral administration of *S. officinalis* extract. To our best knowledge, the pharmacokinetics study of the DGE, AGE, RMU, HDA and APS in rats is firstly reported, which will provide the pharmacokinetic rationale for the pharmacology of the DGE, AGE, RMU, HDA and APS. This paper may be useful for more in depth studies on the absorption process of *S. officinalis* extract in vivo as well as beneficial for application of this TCM in clinical therapy.

## Figures and Tables

**Table 1:** Mass spectrometric parameters of six compounds and IS.

Compounds	Precursor Ion (*m*/*z*)	Product Ion (*m*/*z*)	Fragment (V)	Collision Energy (V)	Polarity
**I**	784.5	437.4	150	10	Positive
**II**	652.5	455.4	130	20	Positive
**III**	771.5	609.1	300	48	Positive
**IV**	673.4	511.4	330	40	Positive
**V**	505.2	423.2	110	15	Positive
**VI**	655.4	493.0	290	34	Positive
**VII**	418.9	342.8	78	18	Positive

**Table 2:** Matrix effects and extraction recoveries for analytes and IS in rat plasma (*n* = 6).

Compound	Spiked Concentration (ng/mL)	Matrix Effect (%)		Extraction Recovery	
		Mean (%)	RSD (%)	Mean (%)	RSD (%)
**I**	12.1	95.47	3.87	79.06	4.71
	121.0	97.78	6.31	81.80	8.79
	1936	100.17	9.24	86.17	3.22
**II**	9.8	92.93	3.87	81.11	5.84
	97.5	97.60	6.93	83.08	11.22
	1560	101.30	10.88	88.18	3.79
**III**	2.7	93.77	6.63	77.17	11.52
	26.7	98.05	5.69	86.55	3.90
	426.6	99.64	6.03	91.80	2.58
**IV**	7.6	92.03	7.52	79.91	8.25
	75.5	96.96	6.14	84.33	6.17
	1208	102.10	9.44	89.59	4.68
**V**	3.0	92.96	3.21	80.57	11.06
	30.2	95.96	4.57	84.06	10.78
	483.2	101.69	8.55	90.03	8.71
**VI**	11.3	96.03	4.14	77.81	14.32
	112.5	98.95	6.09	86.76	10.98
	1800	100.31	8.39	88.79	4.93
**VII**	1040	95.16	4.72	92.48	6.87

**Table 3:** The regression equations, linear ranges, LLOD_S_ and LLOQs for the determination of analytes in rat plasma (*n* = 7).

Compound	Regression Equation	*r*	Linear Range (ng/mL)	LLOQ (ng/mL)	LLOD (ng/mL)
**I**	*Y* = 2.200 × 10^−3^*X* + 1.771 × 10^−1^	0.9912	6.05–2420	6.05	2.02
**II**	*Y* = 3.310 × 10^−3^*X* + 2.592 × 10^−1^	0.9961	4.88–1950	4.88	1.63
**III**	*Y* = 5.688 × 10^−4^*X* + 4.224 × 10^−2^	0.9954	1.34–533.3	1.34	0.45
**IV**	*Y* = 2.600 × 10^−3^*X* + 9.874 × 10^−2^	0.9958	3.78–1510	3.78	1.26
**V**	*Y* = 1.010 × 10^−3^*X* + 2.789 × 10^−2^	0.9928	1.51–604.0	1.51	0.50
**VI**	*Y* = 1.600 × 10^−4^*X* + 3.860 × 10^−3^	0.9981	5.65–2250	5.65	1.88

**Table 4:** Intra-day and inter-day precisions and accuracies for the determination of six analytes in rat plasma (*n* = 6).

Compound	Nominal Concentration (ng/mL)	Measured Concentration (ng/mL)	Accuracy (RE%)	Intra-Day Precision (RSD%)	Inter-Day Precision (RSD%)
**I**	6.1	5.96 ± 0.38	−8.41	6.49	4.73
	12.1	11.87± 0.65	5.87	5.26	7.01
	121.0	119.61 ± 4.87	−8.15	4.30	6.63
	1936	1930.77 ± 17.76	−4.27	5.85	1.32
**II**	4.9	4.72 ± 0.21	6.21	4.68	3.14
	9.8	9.66 ± 0.47	3.89	4.20	8.07
	97.5	95.07 ± 7.70	−7.50	7.33	12.42
	1560	1500.56 ± 62.15	−3.81	3.84	5.92
**III**	1.3	1.33 ± 0.08	7.83	6.01	3.64
	2.7	2.66 ± 0.26	−6.29	10.11	6.37
	26.7	25.81 ± 1.13	−3.26	4.46	3.57
	426.6	421.06 ± 10.59	−7.30	4.60	7.75
**IV**	3.8	3.75 ± 0.46	5.73	12.74	7.65
	7.6	7.39 ± 0.74	−8.14	10.42	6.66
	75.5	73.28 ± 7.58	−6.94	10.96	3.07
	1208	1214.24 ± 28.08	3.52	6.82	4.54
**V**	1.5	1.48 ± 0.16	6.06	11.28	7.18
	3.0	2.95 ± 0.28	−9.48	10.02	4.13
	30.2	29.13 ± 2.79	−3.53	9.97	5.63
	483.2	470.38 ± 27.72	−8.65	6.06	4.46
**VI**	5.7	5.91 ± 0.35	4.53	6.26	2.65
	11.3	11.71 ± 0.70	10.15	5.31	9.51
	112.5	119.44 ± 9.85	6.17	7.83	10.83
	1800	1831.19 ± 45.05	2.73	5.52	8.93

**Table 5:** Stability of six analytes in rat plasma under various conditions (*n* = 6).

Compound	Spiked Concentration (ng/mL)	Stability (% RE ^a^)			
		Short-Term	Long-Term	Three Freeze-Thaw	Post-Preparation
**I**	12.1	4.27	2.43	6.07	−2.07
	121.0	6.27	−6.57	10.19	3.53
	1936	2.44	2.03	2.51	−9.95
**II**	9.8	−5.94	−3.19	2.66	5.22
	97.5	4.60	2.08	−2.95	4.11
	1560	2.47	2.24	2.74	3.04
**III**	2.7	−5.54	3.75	−7.72	−1.34
	26.7	−7.66	−5.18	−5.29	−2.48
	426.6	−2.42	4.31	−3.38	1.10
**IV**	7.6	9.63	4.88	6.80	7.45
	75.5	7.81	2.71	3.84	2.23
	1208	4.55	−6.75	2.07	3.95
**V**	3.0	−4.77	9.86	−9.67	8.48
	30.2	−3.67	6.95	3.99	5.23
	483.2	2.56	3.06	2.28	−3.03
**VI**	11.3	−10.78	−2.07	2.74	3.18
	112.5	2.34	2.79	6.61	9.78
	1800	3.26	5.28	−1.64	4.85

^a^ RE is expressed as: (measured concentration-spiked concentration)/spiked concentration × 100%.

**Table 6:** Mean plasma concentration-time curve of six analytes in rats after oral administration of *S. officinalis* extract (*n* = 12, mean ± SD).

Compounds	*C_max_* (ng/mL)	*T_max_* (h)	*t*_1/2_ (h)	AUC_0__→t_ (ng·h/mL)	AUC_0__→__∞_ (ng·h/mL)
**I**	744.7 ± 85.7	0.92 ± 0.20	11.63 ± 3.07	2879.96 ± 303.36	3319.05 ± 429.07
**II**	526.6 ± 64.0	0.83 ± 0.26	8.99 ± 3.04	2296.46 ± 416.63	2661.61 ± 600.92
**III**	317.9 ± 47.6	1.33 ± 0.26	6.86 ± 2.91	1139.15 ± 150.38	1231.82 ± 192.74
**IV**	314.5 ± 22.5	1.58 ± 0.20	7.32 ± 2.74	1208.39 ± 119.71	1302.86 ± 192.89
**V**	211.5 ± 11.8	0.58 ± 0.20	7.35 ± 3.95	733.31 ± 94.08	794.41 ± 151.77
**VI**	243.2 ± 19.9	1.17 ± 0.26	7.72 ± 1.22	1026.03 ± 73.43	1112.72 ± 98.12

## Supplementary Materials

The following are available online at http://www.mdpi.com/1420-3049/23/11/2980/s1, Table S1: The elution program of six compounds, Figure S1: The HPLC-ELSD diagram of six compounds in *Sanguisorba officinalis* L. extract.

## Abbreviations

*Sanguisorba officinalis* L.	*S. officinalis*
Ziyuglycoside I	ZGI
3β,19α-dihydroxyurs-12-en-28-oic-acid 28-β-d-glucopyranosyl ester	DGE
3β-[(α-l-arabinopyranosyl) oxy]-urs-12,18(19)-dien-28-oic acid β-d-glucopyranosyl ester	AGE
Rosamultin	RMU
1β-hydroxyeuscaphic acid	HDA
Alpinoside	APS
Multiple reaction monitoring	MRM
d-galactosamine	d-GalN
Electrospray ionization	ESI
Quality control	QC
Lower limits of quantification	LLOQ
Lower limit of detection	LLOD
Solide Phase Extraction	SPE
Liquid liquid extraction	LLE
Relative standard deviation	RSD
