# Supplementary material for: Simultaneous Determination and Pharmacokinetics Study of Six Triterpenes in Rat Plasma by UHPLC-MS/MS after Oral Administration of Sanguisorba officinalis L. Extract
Source: Molecules. 2018 Nov 15;23(11):2980. doi: 10.3390/molecules23112980 (PMC6278537; doi:10.3390/molecules23112980)
Supplement: Supplementary file 1 [file molecules-23-02980-s001.pdf]

Simultaneous determination and pharmacokinetics study of six triterpenes in rat plasma by UHPLC-MS/MS after oral administration of *Sanguisorba officinalis* L. extract

Chengcui Wu [1], Meicun Yao [2], Wa Li [2], Binbin Cui [1], Hongrui Dong [1], Yixuan Ren [1], Chunjuan Yang [1,*] and Chunli Gan [3,*]

1. Simultaneous determination of six triterpenes from *Sanguisorba officinalis* L. extract by HPLC-ELSD

Table S1: The elution program of six compounds.

Time（min）	Water(%)	Acetonitrile(%)
0	90	10
20	60	40
30	50	50
45	10	90
50	10	90
60	90	10

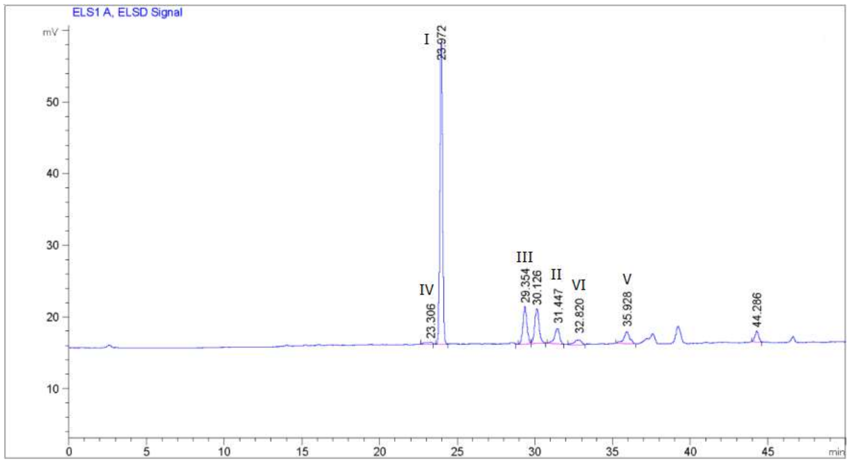

Figure S1: The HPLC-ELSD diagram of six compounds in *Sanguisorba officinalis* L. extract
